# Biodegradable Magnesium (Mg) Implantation Does Not Impose Related Metabolic Disorders in Rats with Chronic Renal Failure

**DOI:** 10.1038/srep26341

**Published:** 2016-05-23

**Authors:** Jiali Wang, Jiankun Xu, Waiching Liu, Yangde Li, Ling Qin

**Affiliations:** 1 Musculoskeletal Research Laboratory, Department of Orthopaedics & Traumatology, The Chinese University of Hong Kong, Hong Kong SAR, P.R. China; 2 Center for Translational Medicine Research and Development, Institute of Biomedical and Health Engineering, Chinese Academy of Sciences, Shenzhen 518055, P.R. China; 3 Guangdong Innovation Team for Biodegradable Magnesium and Medical Implants, E-ande Dongguan 523660, P.R. China

## Abstract

Mg and its alloys have been considered as one of the most promising biodegradable medical devices, but it was still unclear whether hypermagnesemia involved health risks would occur in persons with kidney disease due to their deteriorated kidney function for Mg ions excretion from their body. In this study, we established a chronic renal failure (CRF) model in rats induced by adenine administration prior to Mg implantation, aiming to predict if CRF patients are suitable for the use of Mg implants. The results showed that Mg levels in serum, urine, feces and internal organs had no significant changes after Mg implantation for both normal and CRF rats. Biochemical indices detection and histopathological analysis in kidney, liver and heart tissue confirmed that Mg implants did not induce any extra damage in animals even with renal failure. Our study indicates that Mg based orthopaedic medical device may be considered for use in CRF patients without biosafety concerns.

Magnesium (Mg) or its based alloys have been recognized as the novel generation of biometals suitable for developing cardiovascular stents or bone fracture fixators attributed to their advantages of biodegradability, appropriate mechanical strength or modulus without inducing stress shielding, osteopromotive effects, bacterial inhibition and no concerns of artifacts in diagnosis imaging over current inert metallic counterparts^1^,^2^,^3^,^4^,^5^,^6^. In orthopaedics, the release of Mg ions from the Mg implants via the attack of chloride ions (Cl^−^) and ingestion of macrophages in Mg-based biodegraded products could effectively promote bone formation^7^,^8^,^9^,^10^,^11^. The underlying mechanism to stimulate the acceleration of bone fracture healing may be linked with the positive contributions to osteogenic differentiation of stem cells and angiogenesis of endothelial cells while inhibitory effects on osteoclast function in the presence of increasing Mg level^9^,^12^,^13^. Currently, multiple animal species including mouse, rat, guinea pig, rabbit and sheep have been used to establish various preclinical models to mimic relevant clinic indications, i.e. avulsion fracture and open fracture in load-free or heavy-weight bearing parts, providing a prescreening information and also paving the way for the following clinic study^14^,^15^,^16^,^17^. Actually, a huge progress of R&D in Mg implants has been made as the pilot study regarding the treatment of patients by using Mg medical devices in Germany, i.e. 13 patients for hallus valgus surgery with 6-month follow up observation^18^, China, i.e. 23 patients for femoral head osteonecrosis treatment with 12-month follow up observation^19^, and Korea^20^, i.e. 53 cases with over 1 year, showed encouraging clinic outcomes.

All these above achievements truly indicate that the translational work of such biodegradable metals may be soon applied in the clinic trials and improve the healing quality of bone fracture. However, we have to keep in mind that all these scientific data regarding the use of Mg implants were acquired from the healthy animal models or patients without metabolism disorders, suggesting that the potential health risks induced by the released Mg ions from the implants in special groups with metabolic organ dysfunction have not been ever considered or evaluated. The total Mg level in the serum of adult was between 0.65 and 1.05 mM; and only if serum Mg level exceeded 3.5 mM, signs of mild Mg toxicity would appear, including hypotension, cardiac arrhythmias, or bradycardia etc.^21^. For healthy individuals, the excessive Mg ions could be effectively excreted from the body via urine and feces to keep Mg balance in the plasma^21^. In fact, previous clinical studies in patients using Mg-based fixators have confirmed their biosafety^18^,^22^,^23^,^24^. Besides, the direct administration of Mg sulfate agent via intramuscular and intravenous injection into patients was also widely used as a clinic therapy for preventing or treating eclampsia, focal cerebral ischemia and stroke^25^,^26^,^27^. The immediate given dose of Mg sulfate by the combined intramuscular and intravenous routes can sometimes reach 14 g, but up to 90% of Mg ions would be eliminated within the first 24 hours via urine excretion^25^. However, we have to keep in mind that it is the normal kidney function to facilitate the Mg homeostasis via excretion by glomeruli and reabsorption by tubular. Once the kidney function is damaged, the patients may have higher risks to suffer hypermagnesemia if the degradation products could not be excreted from their body smoothly. Actually, it has been reported that severe side-effects caused by hypermagnesemia might be induced in the presence of renal failure as the reduction in the glomerular filtration rate (GFR) contributes to the impaired function of kidney with regards to Mg elimination from blood, causing higher serum Mg ion concentration^21^. For the patients especially with the end-stage chronic renal failure, the long term dialysis may easily induce disordered Mg homeostasis^28^,^29^. Approximately, it is estimated that 17% of the adult population was suffering from chronic renal diseases at various progressive stages^30^, so it is very urgent and important for us to individually evaluate the potential health risks of Mg implants in these special groups. If we have not sufficient evidence for the assurance of the biosafety for the use of Mg based medical devices in the patients with the deteriorated kidney function, we have to keep cautious to select target population and provide comments to clinicians on how to define the potential contraindications for the use of biodegradable Mg implants. From this aspect, our work targeted one of the most crucial elements required for biomaterials, which is biosafety prior to efficacy tests.

However, how to establish a reliable animal model to mimic the chronic renal failure in patients is a crucial factor influencing our ultimate evaluation. As the underlying mechanisms contributing to the chronic renal failure progression vary in individuals^31^, it is more appropriate for us to establish an animal model with similar end-point indices to patients. Currently, 5/6 nephrectomy used for studying glomerulosclerosis and unilateral ureteral obstruction (UUO) aimed for tubulointerstitial fibrosis were the two most common models in rats to study the impaired function in chronic kidney disease^32^. However, a complex process including glomerular filtration, tubular excretion and tubular reabsorption was involved in urine excretion^33^. In this study, we adopted a non-surgical model by using oral adenine administration in rats to induce pathological changes of kidney with both impaired glomerular and tubular function to mimic patients with chronic renal failure (CRF), aiming to assess the effects of Mg implants on their health risks^34^. We hypothesized that the degradation of Mg implants would not further deteriorate the kidney function in CRF group with respect to biochemical indices in serum and histopathological analysis in tissue.

## Results

### Features of CRF, measurement of biochemical indices, and Mg metabolism analysis

**Figure 1:**
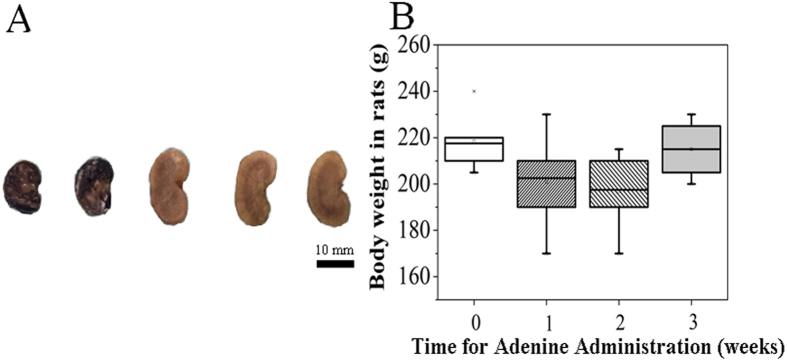
Morphological changes of kidney and body weight changes of rats after oral administration of adenine. (**A**) Kidneys were fixed with formalin and dehydrated (left to right: Control group at week 2 versus surgical time; Mg group at week 2 versus surgical time; CRF group at week 3 after adenine administration prior to surgery; CRF and CRF-Mg groups at week 2 versus surgical time; (**B**) Temporal changes of body weight (BW) in rats after oral adenine administration within 3 weeks in CRF group (n = 6). Scale bar, 10 mm.

**Figure 2:**
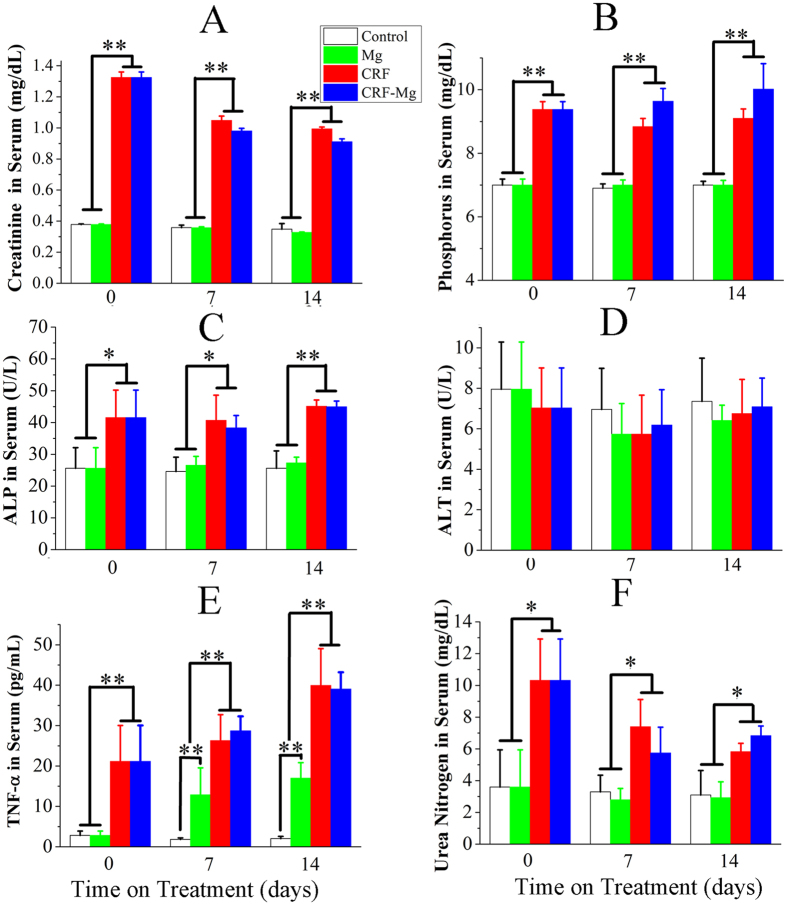
Temporal changes of biochemical indices in serum of rats in Control, Mg, CRF, and CRF-Mg groups. (**A**) Creatinine; (**B**) Phosphorus; (**C**) ALP; (**D**) ALT; (**E**) TNF-α and (**F**) Urea nitrogen. Control group or Mg group at week 0 versus surgical time stands for the rats with MC administration for 3 weeks prior to surgery while CRF or CRF-Mg group at week 0 represents the rats with adenine administration for 3 weeks prior to surgery. *p < 0.05, **p < 0.01, n = 6.

**Figure 3:**
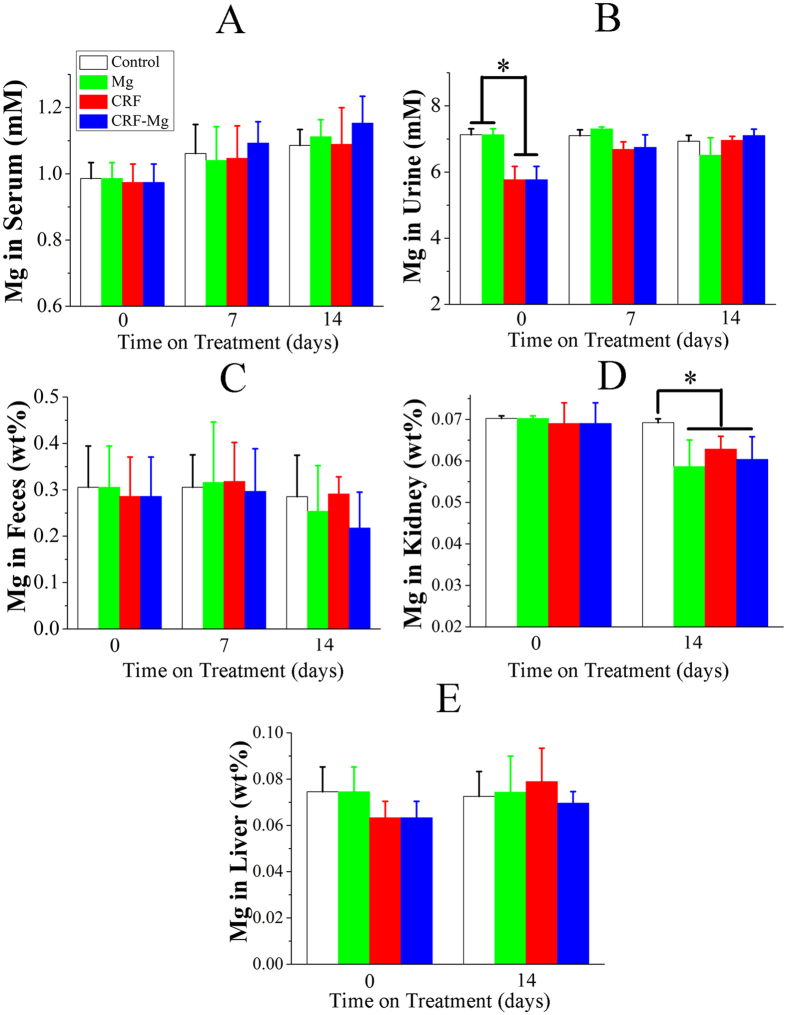
Temporal changes of Mg concentrations in (**A**) Serum, (**B**) Urine, (**C**) Feces, (**D**) Kidney, and (**E**) Liver in rats of Control, Mg, CRF and CRF-Mg groups. Control group or Mg group at week 0 versus surgical time stands for the rats with MC administration for 3 weeks prior to surgery while CRF or CRF-Mg group at week 0 represents the rats with adenine administration for 3 weeks prior to surgery. *p < 0.05, n = 6.

After an oral administration of 0.75% adenine solution in rats for 3 weeks, we observed that the size of kidney enlarged and the color changed from dark red to yellowish white (Fig. 1A). However, the implantation of Mg pins did not cause additional changes in size and color in kidney tissue in both Mg group and CRF-Mg group. Interestingly, no significant changes of body weight were detected during the entire experimental period (Fig. 1B). After 3 weeks of oral administration of adenine, serum levels of creatinine, phosphorus, ALP, TNF-α and urea nitrogen in CRF rats rose significantly (Fig. 2A–C,E,F). The intramedullary implantation of Mg pins did not induce remarkable changes in the five biochemical indices in both CRF-Mg and Mg groups within the 2 weeks (Fig. 2A–C,E). ALT level in serum of rats was not affected by adenine administration and Mg implantation (Fig. 2D). For healthy rats, the implantation of Mg pins did not significantly increase Mg amount in serum, urine, feces, kidney and liver tissue during the entire experimental period (Fig. 3). The adenine administration in rats did not remarkably affect Mg levels in serum, urine, feces and internal organs. After CRF induction in rats, we did not find any temporal changes of Mg dose in serum, urine, feces, kidney and liver even in the presence of Mg pins insertion (Fig. 3).

### Histological analysis

**Figure 4:**
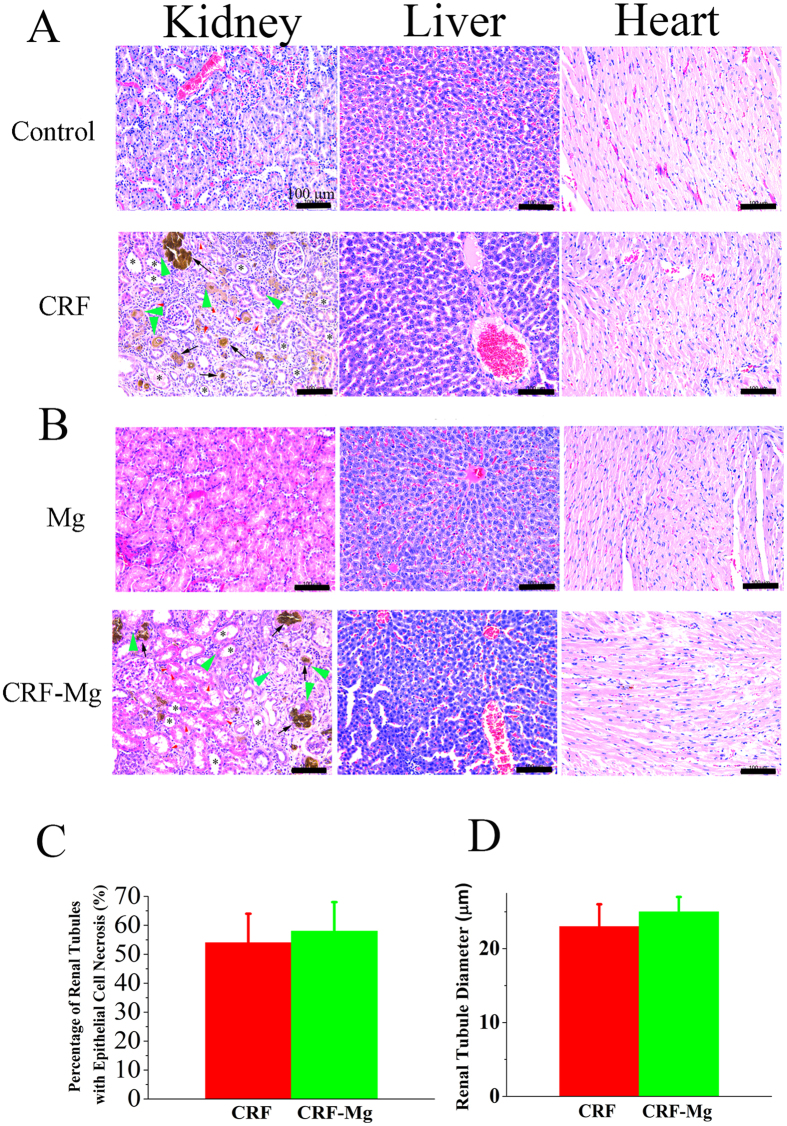
Histological findings of organs harvested from rats at 2 weeks postoperatively. (**A**) H&E staining of kidney, liver and heart of rats in Control and CRF groups at week 2 versus surgical time; (**B**) H&E staining of kidney, liver and heart of normal and rats in Mg and CRF-Mg groups at week 2 versus surgical time; (**C**) Comparison of percentage of injured tubules out of all tubules in kidney of rats between CRF and CRF-Mg groups at week 2 versus surgical time (n = 4) and (**D**) Comparison of renal tubular diameters in rats between CRF and CRF-Mg groups at week 2 versus surgical time (n = 4). Crystals (arrows), large dilated tubules (asterisks), granuloma (green arrowheads) and epithelial cell necrosis (red arrowheads) in CRF kidney induced by adenine were labeled. Scale bar, 100 μm.

The administration of adenine caused the deposition of 2,8-dihydroxyadenine crystals in the kidney and induced formation of granulomas in the renal tubules and interstitium (Fig. 4A). Mild dilatation of renal tubules was observed due to the occupation of crystals or granulomas, which was accompanied with epithelial cell necrosis (Fig. 4A). The absence of kidney damage involved pathological features was observed in Mg implanted healthy rats (Fig. 4B). More importantly, the biodegradation products from Mg pins did not induce further deterioration in kidney tissue of CRF rats via the comparison of the percentage of the injured renal tubules and enlargement in renal tubule diameter between CRF group and CRF-Mg group (Fig. 4C,D). Besides, we did not observe any pathological signs of heart and liver disease via H&E staining analysis after the insertion of Mg pins into healthy and CRF rats within the entire experimental period (Fig. 4B).

### IHC analysis for CD68 positive cells

**Figure 5:**
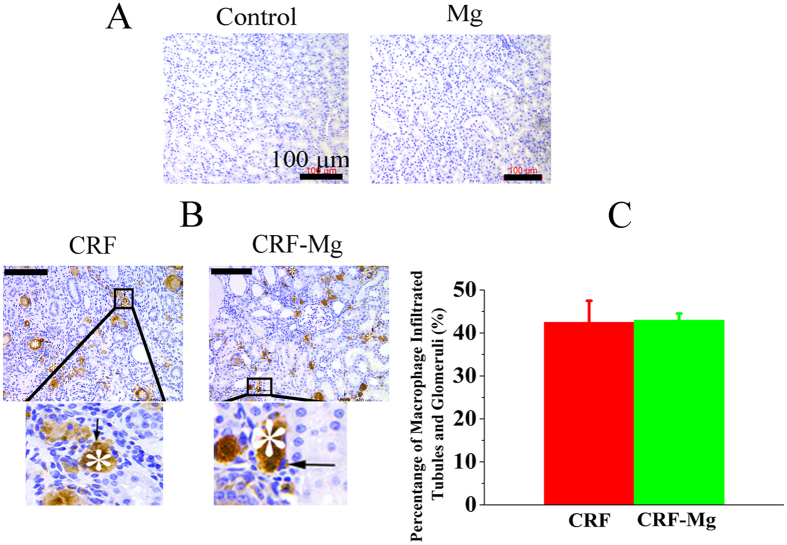
Immunohistological staining of kidney for detection of CD68 positive cells (arrows) in rats of Control, Mg, CRF, and CRF-Mg groups at week 2 versus surgical time. (**A**) Control and Mg groups; (**B**) CRF and CRF-Mg groups and (**C**) Statistical analysis of percentage of renal tubules and glomeruli infiltrated with macrophages expressing CD68 in kidney of rats of CRF and CRF-Mg groups (n = 4). Crystals were labeled with asterisks. Scale bar, 100 μm.

Mg implants did not induce aggregation of macrophages in the kidney of healthy rats (Fig. 5A). However, the presence of macrophages in the kidney of CRF rats was confirmed by immunostaining with anti-rat macrophage CD68 polyclonal antibody. Most of the CD68 positive cells were observed around the crystals, but the implantation of Mg pins did not induce significant rise of number of macrophage infiltrated tubules and glomeruli in CRF rats (Fig. 5B,C).

### Discussion

Biosafety evaluation is necessary for any candidates of Class III medical devices prior to their use mandated by regulatory agencies in clinic study. The pioneer research of Mg based implants as orthopaedic devices dated back to one century ago^22^. However, due to limitations in metallurgical and related processing technology, low Mg purity and related crystal defects remained abundant in commercial Mg material, leading to undesirable faster degradation at early phase and therefore no sufficient mechanical support for fracture fixation and stable healing^22^. The purity improvement in Mg metal matrix was one of the most important and cost-effective strategies to modify the corrosion resistance of Mg based medical devices. In this study, we fabricated Mg implants with high purity, aiming to explore their use potential in target population with or without metabolism disorders. As one of the most serious metabolic organ diseases, kidney failure in patients may induce higher risks in the presence of biodegradable Mg implants due to deteriorated kidney function for ions clearance. Herein, we established chronic renal failure model in rats to mimic relevant clinic indication to test if any hypermagnesemia involved risks would occur during the Mg implantation period, aiming to provide the proof for the clinic study. Our findings based on the short-term *in vivo* study indicated that the use of Mg based implants may not induce extra health risks in patients with kidney diseases.

In order to evaluate the potential health risks induced by degradation products from Mg implants in those patients with deteriorated kidney function, we established chronic renal failure model in rats by adenine administration. Briefly, the induction of chronic renal failure by adenine feeding is dependent on the enzymatic production of an adenine metabolite, 2,8-dihydroxyadenine, and its deposition in the kidney. After 3 weeks of administration of adenine, the accumulation of crystals in renal tubules and glomeruli seriously deteriorated the kidney function as the level of serum creatinine (from 0.37 to 1.3 mg/dL) and phosphorus (from 7 to approximate 9 mg/dL) rose significantly^35^,^36^. Besides the two most important indicators for prediction of kidney failure, ALP, urea nitrogen and TNF-alpha concentrations in serum also showed remarkably increase, suggesting pathological changes of rat kidney. The presence of a large number of necrotic epithelial cells and macrophage infiltrated renal tubules and glomeruli further confirmed that the chronic renal disorder model was successfully established. In the following 2 weeks after CRF model establishment, the pathological signs of kidney failure was still present according to biochemical indices measurement and histopathological analysis, indicating the effectiveness for our adoption of adenine induced CRF model during the while experimental period. Although GFR was not measured in this study, the early stage of kidney disease could be diagnosed in these rats according to the reported data in related publications^36^,^37^,^38^.

Up to now, none of hypermagnesemia involved incidences have been reported in animals or patients, who were performed surgery by using Mg based medical devices in the absence of kidney disease. Generally, the released Mg ions could be effectively excreted from body via urine and feces or absorbed by surrounding tissue for realizing its biological function(s), e.g. as co-enzyme or biological signal element for accelerating new bone formation^18^,^39^. However, the biosafety concerns regarding the use of biodegradable Mg metals in special population, who are suffering from chronic disease in metabolic organs including liver and kidney, will have to be raised due to their reduced clearance capability of excessive metal ions^40^. In this study, we evaluated the health risks of Mg based implants in CRF rats that formed a foundation of pre-screening for applying biodegradable medical implants in patients with kidney dysfunction. After implantation of Mg pins in CRF rats, we did not observe the occurrence of abnormal behavior in animals, indicating tolerable for degradation products during the experimental period. Meanwhile, we performed the degradation rate measurement of pure Mg pins by using both *in vitro* and *in vivo* testing models and found Mg corroded fastest in the initial stage (Supplementary Fig. S1). Our pilot *in vivo* study showed the corrosion rate of Mg pins in the first 2 weeks was 5-folder higher than that in the following 6 weeks (0.821 ± 0.094 mm/yr (10% volume loss) vs. 0.130 ± 0.024 mm/yr (5% volume loss)), indicating the highest risk for the rapid accumulation of Mg ions in the body within the first 2 weeks if the excretion function in kidney was seriously deteriorated (Supplementary Fig. S1). Therefore, we selected the first 2 weeks to detect the most relevant biochemical indices in serum and perform relevant histopathological examinations for the kidney. Interestingly, the implantation of Mg pins did not trigger a significant rise of Mg level in serum, internal organs including liver and kidney and excrement, i.e. urine and feces in both normal and CRF rats during the whole period. Considering that Mg ions could significantly promote new bone formation around periosteal region, partial released Mg ions might be explored for tissue regeneration. Actually, more Mg content was incorporated in the newly formed tissue adjacent to the implants as higher intensity assigned for Mg signal was detected via energy dispersive x-ray spectroscopy (EDS) analysis (Supplementary Fig. S2A), which was also supported by our *in vitro* findings via the detection of higher Mg amount in calcium nodules secreted by bone marrow stem cells (BMSCs) cultured in osteogenic medium with rising Mg levels (Supplementary Fig. S3). The biodegradation products mainly composed of Mg(OH)_2_, MgCO_3_ and Mg_3_(PO_4_)_2_ from Mg implants showed similar chemical composition to the calcified matrix in native bone (Supplementary Fig. S2B–D), which may facilitate biological integration of Mg metals and surrounding bone tissue^20^.Therefore, the store of Mg elements in peri-implant tissue and the use of transported Mg ions for bone regeneration may partially contribute to the flow of released Mg ions from Mg metal in the body^20^. In the present study, only 10% volume of Mg implants was reduced during the entire implantation period, indicating that 0.005 g of Mg was released within the first two weeks (Supplementary Fig. S1). For an adult, the total body storage of Mg was approximately around 25 g and about 66% was stored in bone^41^. As Mg mass accounts for 0.038% in body weight of adults (65 kg as an example), the estimated mass of Mg in rats (220 g) should be 0.085 g. Thus, even in the extreme conditions that the released Mg ions could not be excreted from body, only 5% increase of Mg in bone would be estimated. However, for CRF patients with dialysis treatment, bone Mg was increased over 50% in both cortical and trabecular bones^41^, indicating the tolerable amount for the released Mg ions from implants in the body. Clinically, hypermagnesemia syndrome seldom occurs in CRF patients unless GFR was below 30 ml/min in the presence of high dose administration of Mg agent for treatment^29^,^42^. Actually, the impairment of renal excretion function is always accompanied with the deteriorated ability of tubular reabsorption for Mg depletion even when GFR is very low in stages 3 and 4 CRF^43^, so the ultimate inflow of Mg ions into blood will be effectively compromised.

More importantly, a high frequency of fractures and increased adverse outcomes following a fracture were observed in patients with renal disease^44^, as the abnormal elevated PTH secretion in patients with renal osteodystrophy (ROD) contributes to increased catabolism for cortical bone, leading to deterioration in cortical (micro)architecture and decrease in bone density^45^. However, the increase of Mg concentrations in the body may help to suppress PTH secretion and modulate the rate of bone remodeling, leading to an optimized microarchitecture and mechanical properties of newly formed bone tissue^45^,^46^. Therefore, the use of Mg implants as novel orthopaedic devices might significantly improve bone quality around the fracture site in CRF patients. Most importantly, compared to the general population with bone fracture, CRF patients suffering from fracture have much higher morbidity and mortality^47^. The most common cause was vascular calcification, accounting for 45% of all deaths after bone fracture for CRF patients^44^. As Mg has been widely used as the effective agent for inhibition of apatite deposition in vascular wall, the circulation of the released Mg ions from the implants in the blood might retard the development of vascular calcification and reduce the mortality rate of CRF patients^28^,^48^. Therefore, this would rule out the concerns on biosafety related to accumulation of Mg in body for those patients not suffering from end-stage kidney disease.

In order to validate above analysis and monitor the health condition of normal and CRF rats with Mg implants, biochemical indices as well as histopathological analysis at tissue level were detected in the present study for potential risk prediction prior to the application of Mg orthopaedic devices in CRF patients. As the most commonly used biochemical indices for evaluation of renal function, serum creatinine, phosphorus and ALP level provided sensitive information regarding kidney structure and function^49^. ALT is one of the most important indicators of liver function^50^. No significant changes were observed in these liver and kidney function involved indices for both normal and CRF rats after implantation of Mg pins within the whole experimental period, indicating no impairment to liver and renal function. However, these biochemical indicators might also have their limitations for prediction of function of internal organs especially in the early stage of disease^50^. Histopathological analysis has been widely recognized as the gold standard for diagnosis of disease progression^51^. In this study, the absence of pathological changes in liver, kidney and heart of normal rats after Mg insertion showed that the degradation products were highly biocompatible, which was consistent with the work reported by other team^52^. For CRF rats, the implantation of Mg pins did not induce extra necrosis of epithelial cells and increase macrophage infiltrated tubules and glomeruli in kidney tissue. Besides, none of pathological occurrence in heart and liver tissue was observed in CRF rats after Mg implantation. All the above observation indicated that patients with renal dysfunction might be indicated for use of Mg based implants without concerns of health risk.

However, limitations for this work also exist. Firstly, although our study confirmed the short term biosafety of Mg implants in CRF rats, evaluation with longer experimental time is still required to rule out potential long-term biosafety concerns. Secondly, the progression of kidney disease has not been classified in details in our established chronic renal failure model as no widely recognized standards or guidelines have been proposed to define stages of kidney disease in rats. Similar stages of kidney failure in animals should be established to mimic clinic patients to evaluate their individual responses to Mg implants, aiming to remove the contraindication for patients with corresponding stage of kidney disease.

In summary, our work confirmed that the use of biodegradable Mg medical devices did not trigger health risks in CRF rats via both biochemical indices and histopathological analysis, revealing the application potential of such novel implants as fracture fixators in CRF patients.

## Methods and Materials

### Materials preparation and sterilization

Pure magnesium (99.99% purity with chemical composition shown in Table 1) was prepared by authors’ group through double vacuum distillation process and then remelted for the following extrusion into rods by E-ande corporation in Dongguan, PR China. Cylindrical specimens with 1.2 mm in diameter and 25 mm in length (approximate 0.05 g per pin) were processed and ultrasonically cleaned with absolute acetone and ethanol to reduce organic substances on the surface. Ultraviolet (UV) was used for sterilization of the specimens.

### Establishment of adenine-induced CRF model in rats and implantation of Mg pins

Female adults SD rats were used to induce CRF prior to implantation of pure Mg pins into femoral medullary cavity according to our established experimental protocol approved by the Animal Ethics Committee of the Chinese University of Hong Kong (13/041-MIS-5) in accordance with the guidelines for the ethical treatment of animals. All efforts were made to minimize animal suffering. A total of 36 rats were divided into 4 groups, including normal control without implantation of Mg pins (Control group), normal rats with Mg pin insertion (Mg group), CRF rats without Mg pins (CRF group) and CRF rats with Mg pins (CRF-Mg group). Briefly, the 0.75% (w/w) adenine sulfate suspension in 0.5% methylcellulose (MC) solution was given daily to the rats in CRF and CRF-Mg groups by oral administration (300 mg/kg)^53^. Animals in the control group and Mg group were given orally with an equal volume of 0.5% MC solution instead of the adenine suspension. After 3 weeks of oral administration, the rats of Mg group and CRF-Mg group were anesthetized with ketamine (75 mg/kg) and xylazine (10 mg/kg) for implantation with Mg pins.

### Analysis of biochemical indices

The rats in above 4 groups were hosted in individual metabolism cages for 24 h for separate collection of feces and urine weekly, i.e. at week 0, 1, and 2. Blood of these rats was also collected weekly for isolation of serum via centrifuge at 3000 g for 10 minutes. The feces, serum and acid treated urine were stored at −80 °C before analysis. These rats were sacrificed to harvest liver, kidney and heart at week 3 and 5 after oral administration. Feces, liver and kidney were kept in freeze drier (Labconco, Kansas, USA) for recording their dry weight. Then, the concentrated nitric acid was used to digest the samples for determining Mg ion concentration with the inductively coupled plasma mass spectrometer (ICP-MS, Agilent Technologies, Tokyo, Japan). In terms of serum, besides the measurement of Mg concentrations with ICP-MS, other biochemical indices, including creatinine, urea nitrogen, phosphorus, alanine transaminase (ALT), alkaline phosphatase (ALP), aspartate aminotransferase (AST), and tumor necrosis factor alpha (TNF-α) were also determined with enzymatic kits (Stanbio, Texas, USA).

### Histological analysis

Liver, kidney, and heart of the rats were fixed in 4% buffered formalin for 3 days and then dehydrated with a series of concentrated alcohol (70%, 80%, 95% and 100%) and xylene prior to paraffin embedding and sectioning at 5 μm in thickness for H&E and immunohistochemical (IHC) staining. ImageJ software (version 1.47t, NIH) was used for quantitative analysis of the renal tubular diameter and the number of renal tubular with epithelial cell necrosis.

### IHC Staining for CD68

Macrophages were identified via positive stained CD68 to evaluate the inflammation of the tissues with necrotic cells^54^. Rabbit anti-rat CD68 polyclonal antibody (Abcam, Cambridge, UK) was selected as primary antibody and peroxidase-conjugated goat anti-rabbit lg G (Abcam, Cambridge, UK) was chosen as secondary antibody. Briefly, after deparaffinization and rehydration, tissue sections were treated with 3% hydrogen peroxide in methanol for 30 minutes to block the endogenous peroxidase activity. Then, the protease k solution (Abcam, Cambridge, UK) was used for antigen retrieval under 37 °C prior to non-specific blocking with ultra V protein block (Dako, Glostrup, Denmark) for 5 minutes. After washing in phosphate-buffered saline (PBS), sections were then incubated overnight at 4 °C with primary antibody. After washing in PBS, sections were incubated with 5% goat serum +1% BSA in PBS for non-specific blocking. Finally, sections were treated with secondary antibody for 1 hour and then placed in substitute media containing 3,3′-diaminobenzidine-tetrachloride (Abcam, Cambridge, UK) before hematoxylin nuclear staining. Image analysis was performed by using ImageJ (1.47t, NIH, USA) to count the number of tubules and glomeruli with macrophage infiltration.

### Statistical analysis

All continuous data were presented as mean ± standard deviation (SD). Statistical analysis was performed with SPSS 17.0. Differences among groups were analyzed using one-way ANOVA followed by post hoc Tukey’s test or independent samples t-test. *p* < 0.05 was considered statistically significant.

## Additional Information

**How to cite this article**: Wang, J. *et al*. Biodegradable Magnesium (Mg) Implantation Does Not Impose Related Metabolic Disorders in Rats with Chronic Renal Failure. *Sci. Rep.* **6**, 26341; doi: 10.1038/srep26341 (2016).

## Supplementary Material

Supplementary Information

## Figures and Tables

**Table 1:** Chemical composition of Mg with high purity (wt.%).

**Metal**	**Composition percentage in weight (%)**									
	**Si**	**Al**	**Ca**	**Ti**	**Mn**	**Fe**	**Ni**	**Cu**	**Zn**	**Pb**
Mg	0.0012	0.0003	0.0003	<0.0001	0.0007	0.0008	<0.0001	<0.0001	0.0009	0.0001
