# Supplementary material for: Biodegradable Magnesium (Mg) Implantation Does Not Impose Related Metabolic Disorders in Rats with Chronic Renal Failure
Source: Sci Rep. 2016 May 23;6:26341. doi: 10.1038/srep26341 (PMC4876325; doi:10.1038/srep26341)
Supplement: Supplementary Information [file srep26341-s1.docx]

**Biodegradable Magnesium (Mg) Implantation Does Not Impose Related Metabolic Disorders in Rats with Chronic Renal Failure**

Jiali Wang^1, 2, #^, Jiankun Xu^1, #^, Waiching, Liu^1^, Yangde Li^3^, Ling Qin^1, 2, 3^^[[1]](#footnote-1)^*

^1^Musculoskeletal Research Laboratory, Department of Orthopaedics & Traumatology, The Chinese University of Hong Kong, Hong Kong SAR, PR China

^2^Center for Translational Medicine Research and Development, Institute of Biomedical and Health Engineering, Chinese Academy of Sciences, Shenzhen 518055, PR China

^3^Guangdong Innovation Team for Biodegradable Magnesium and Medical Implants, E-ande Dongguan 523660, PR China

**Supplementary Figures**


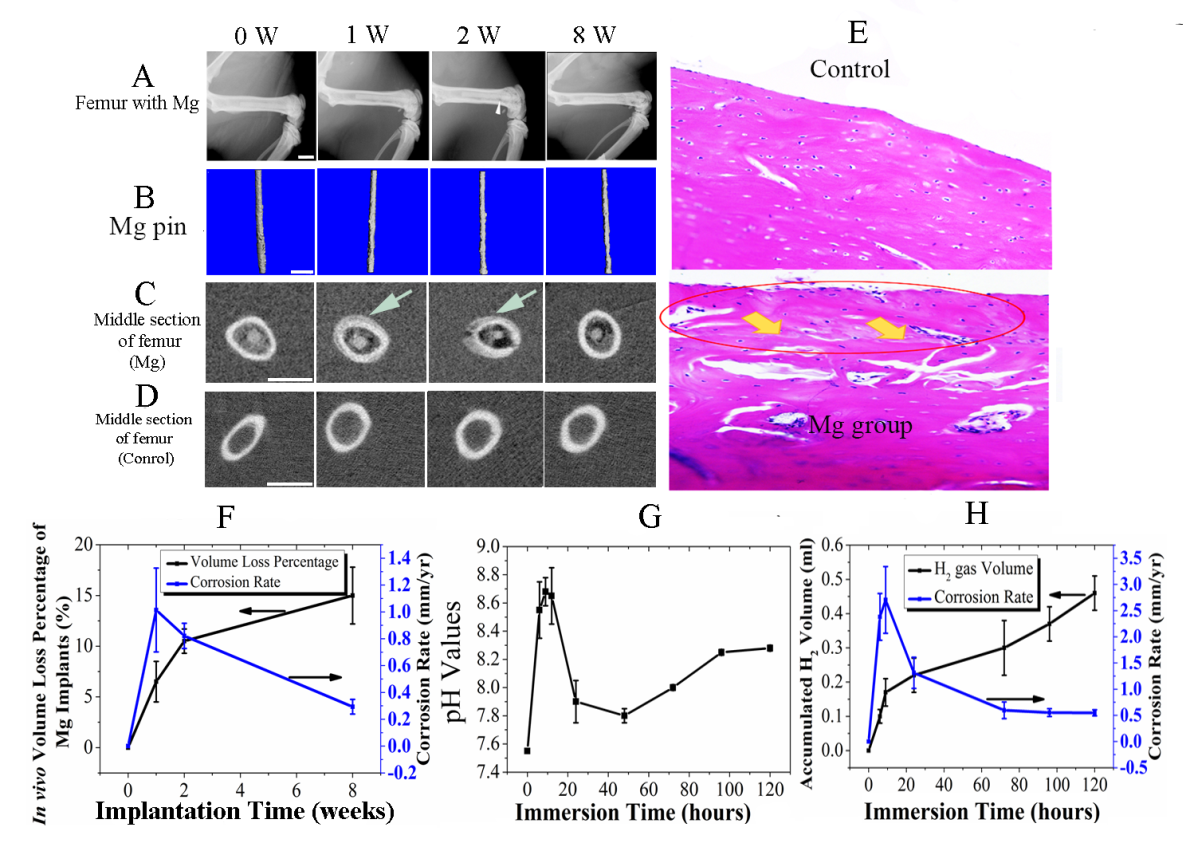


Supplementary Fig. 1 *In vitro* and *in vivo* in degradation rate measurement of Mg pins and involved bone tissue responses after Mg implantation. (A) X-ray imaging of Mg pin inserted femur in rats at week 0, 1, 2, and 8 after surgery. (B) 3D reconstruction imaging of Mg pins. (C-D) 2D tomography images of middle section in femur of rats. Periosteal responses (indicated by arrows) were greatly triggered by the degradation of Mg pins while reduced as the degradation rate slowed down in the late stage. (E) H&E staining of femur in rats with (Mg) and without (Control) Mg implantation. More bone formation labeled by red circle and arrow was detected around the periosteum. (F) *In vivo* degradation rate of Mg pins via CT imaging analysis. The corrosion rate of Mg pins (1.2 mm in diameter and 25 mm in length) in the first 2 weeks was 5-folder higher than that in the following 6 weeks (0.821 ± 0.094 mm/yr (10% volume loss) vs. 0.130 ± 0.024 mm/yr (5% volume loss)). (G-H) Mg pins (1.2 mm in diameter and 10 mm in length) were immersed in cell culture medium (Dulbecco’s modified eagle medium) prior to placement in the CO_2_ incubator for *in vitro* degradation testing via pH and hydrogen gas evolution measurements. An abrupt rise of pH values in medium and more rapid release of hydrogen gas was detected in the initial immersion time for Mg pins. n=3.


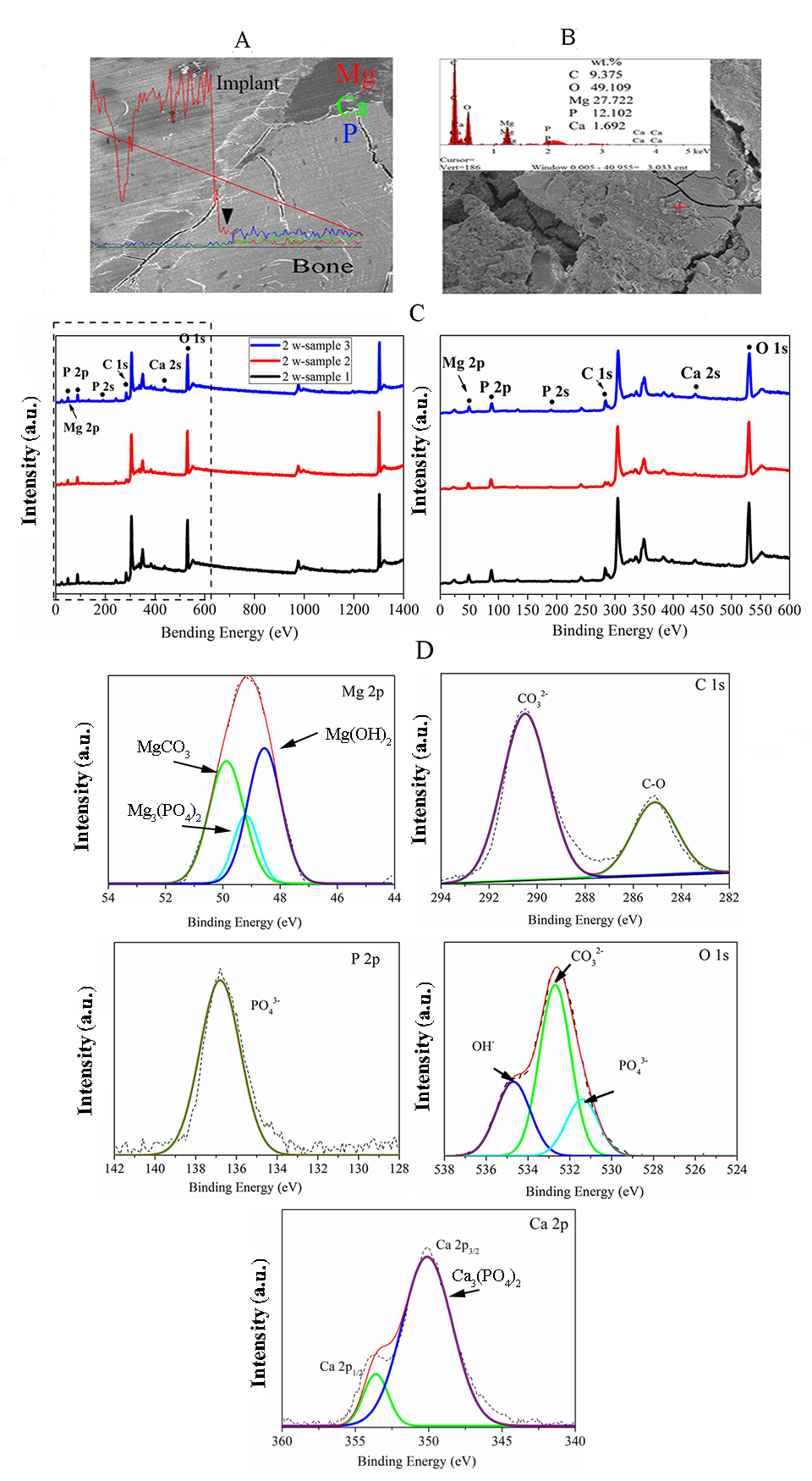


Supplementary Fig. S2 Chemical characterization of degradation products and interface between Mg implants and bone tissue. (A) Elemental distribution analysis along the direction across the interface between Mg and surrounding bone tissue via energy dispersive x-ray spectroscopy (EDS) determination. Higher intensity assigned for Mg peak was observed in the surrounding bone tissue around Mg pins. (B) Elemental composition analysis in degradation products deposited on Mg metal after 2 weeks implantation in rats. (C) An XPS survey scan of pure Mg pins after inserted into bone marrow cavity of femur in rats for 2 weeks (the right picture was the magnified image of the dotted line labeled region in the left picture). (D) High resolution of XPS spectra for Mg 2p, Ca 2p, C 1s, P 2p and O 1s information. Mg involved carbonate, phosphate and hydroxide salts were considered the main constitutes in degradation products. n=3.


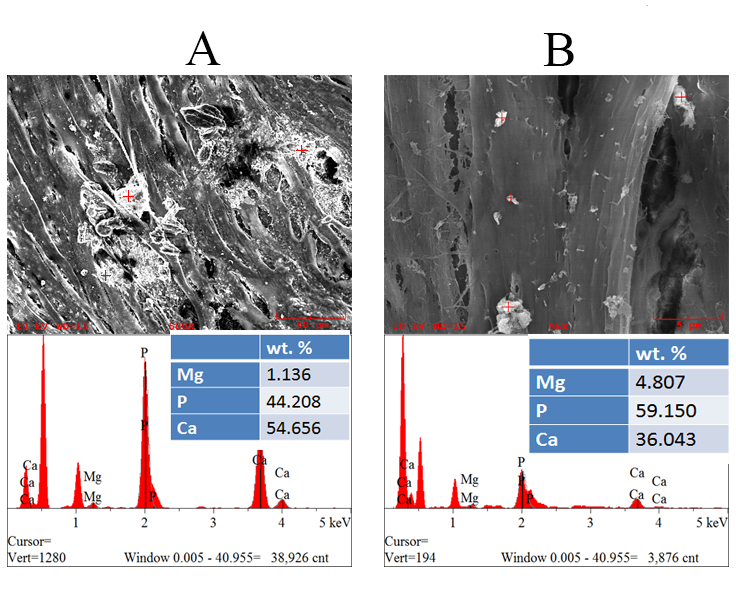


Supplementary Fig. S3 Element composition of extracellular mineral nodules (indicated by red markers) secreted by bone marrow stem cells (BMSCs) from rats after osteogenic differentiation for 3 weeks in osteogenic medium with normal (0.8 mM) (A) and 2 mM (B) Mg ion level. Increasing Mg ion concentration in the osteogenic medium contributes to higher Mg contents in the mineral nodules. 6 nodules in each group were used for the calculation of average contents of target elements including Mg, P and Ca.

1. *Corresponding author. Musculoskeletal Research Laboratory, Department of Orthopaedics & Traumatology, Room 74034, Prince of Wales Hospital, The Chinese University of Hong Kong, Hong Kong SAR, PR China. Tel: +852-26323071; fax: +852-26324618.

   *E-mail address*: [qin@ort.cuhk.edu.hk](mailto:qin@ort.cuhk.edu.hk).

   ^#^The authors contributed equally to this work. [↑](#footnote-ref-1)
